# Engineering Glucose-to-Glycerol Pathway in *Klebsiella pneumoniae* and Boosting 3-Hydroxypropionic Acid Production Through CRISPR Interference

**DOI:** 10.3389/fbioe.2022.908431

**Published:** 2022-06-30

**Authors:** Hexin Liu, Peng Zhao, Pingfang Tian

**Affiliations:** ^1^ College of Life Science and Technology, Beijing University of Chemical Technology, Beijing, China; ^2^ College of Bioscience and Resources Environment, Beijing University of Agriculture, Beijing, China

**Keywords:** *Klebsiella pneumoniae*, glucose, glycerol, 3-hydroxypropionic acid, CRISPR interference

## Abstract

The recent decline of the international biodiesel industry has led to decreased production and therefore increased the price of glycerol, which is a major by-product of biodiesel but a substrate for production of 3-hydroxypropionic acid (3-HP), that is, glycerol as a feedstock has no advantage over glucose in price. Hence, we engineered glucose to the glycerol pathway and improved 3-HP production by CRISPR interference (CRISPRi). To begin with, we cloned the genes encoding glycerol 3-phosphate dehydrogenase (*gpd1*) and glycerol 3-phosphatase (*gpp2*) from *Saccharomyces cerevisiae*, which jointly catalyze glucose into glycerol. The genes *gpd1* and *gpp2* were co-expressed in *K. pneumoniae* with the dCas9 gene integrated in genome, and this recombinant strain produced 2 g/L glycerol in the shake flask. To minimize the glucose consumption by competing pathways including the EMP pathway, glycerol oxidation pathway, and by-products pathways, we developed an CRISPRi system in aforementioned recombinant *K*. *pneumoniae* strain to inhibit the expression of the glyceraldehyde-3-phosphate dehydrogenase gene (*gapA*) and 2,3-butanediol production gene (*budA*), resulting in a bi-functional strain harboring both glucose-to-glycerol pathway and CRISPRi system. Reverse transcription and quantitative PCR (RT-qPCR) results showed that this engineered CRISPRi system transcriptionally inhibited *gapA* and *budA* by 82% and 24%, respectively. In shake flask cultivation, this bi-functional strain produced 2.8 g/L glycerol using glucose as the carbon source, which was 46.6% increase compared to the strain without the engineered CRISPRi system. Moreover, this bi-functional strain produced 0.78 g/L 3-HP using glucose as the sole carbon source. In fed-batch cultivation, this bi-functional strain produced 1.77 g/L 3-HP. This study provides insights for co-utilization of distinct carbon sources.

## Introduction

As an isomer of lactic acid, 3-hydroxypropionic acid (3-HP) is chemically active (de Fouchecour et al., 2018; Matsakas et al., 2018). As such, 3-HP is a versatile platform compound from which a variety of economically important chemicals can be derived. Currently, industrial production of 3-HP relies mainly on chemical synthesis (Della Pina et al., 2011). However, chemical synthesis not only relies on non-renewable resources but also leads to excessive by-products and severe environmental pollution (Della Pina et al., 2011). In recent years, microbial fermentation has emerged as an alternative to chemical synthesis. For instance, 3-HP can be produced by diverse wild-type or engineered strains, including *Escherichia coli* (Mohan Raj et al., 2009), *S. cerevisiae* (Semkiv et al., 2017), *Corynebacterium glutamicum* (Chen et al., 2017), *Lactobacillus reuteri* (Luo et al., 2011), and *K. pneumoniae* (Li et al., 2016). In aforementioned host microbes, 3-HP biosynthesis can be roughly divided into three types: glycerol-based (Kumar and Park, 2018), glucose-based (Straathof et al., 2005), and acetate-based 3-HP pathways (Zhao and Tian, 2021). The glycerol-based 3-HP synthesis involves coenzyme A-dependent pathway and coenzyme A-independent pathway (Jers et al., 2019). The CoA-independent 3-HP production from glycerol requires only two-step catalysis by glycerol dehydrogenase (GDH) (EC 4.2.1.30) and acetaldehyde dehydrogenase (ALDH) (EC 1.2.1.3). Therefore, this 3-HP pathway is of great attractiveness. However, the ALDHs in wild-type strains show low activity, and only trace amount of 3-HP can be generated (Raj et al., 2010; Ko et al., 2012). Fortunately, when the *tac* promoter was used to express PuuC, an ALDH native to *K. pneumoniae*, 3-HP was overproduced from glycerol (Li et al., 2016; Zhao et al., 2019). To produce 3-HP from glucose through the CoA-independent pathway, a glucose-to-glycerol pathway needs to be engineered. To do so, it is necessary to clone the genes encoding glycerol 3-phosphate dehydrogenase (GPD1, EC 1.1.1.8) and glycerol 3-phosphatase (GPP2, EC 3.1.3.21) from *S. cerevisiae* (Zheng et al., 2008). In addition, a host strain tolerant to glycerol is required. Of candidate host strains, *K. pneumoniae* is promising in this regard (Zhao et al., 2019), as it can efficiently metabolize glycerol. Although glycerol is the most used carbon source for production of 3-HP (Nguyen-Vo et al., 2018), its price is high due to the recent decline of the biodiesel industry.

In *S. cerevisiae*, glucose is catalyzed into glycerol by GPD1 and GPP2 (Chen et al., 2014; Kildegaard et al., 2016). In wild-type microbes, glucose conversion to glycerol is rather limited and high-level production of 3-HP is thus challenging. The reason behind is that glucose uptake is mainly accomplished by the phosphoenolpyruvate-dependent glucose uptake system (PTS), and glycolysis pathway (EMP) consumes a large amount of glucose (Chen et al., 2017). Moreover, in the presence of glucose, the Crabtree effect inhibits the conversion of glycerol to 3-HP (Joseph et al., 1981). When metabolic pathways were optimized and glucose was used as the carbon source, the maximum 3-HP titer of engineered *E. coli* reached 37.6 g/L (Heo et al., 2019). Despite a lot of studies, the low 3-HP production constrains its commercialization (Ashok et al., 2011). Hence, global regulation is required to improve the conversion rate of glucose to 3-HP.

**FIGURE 1:**
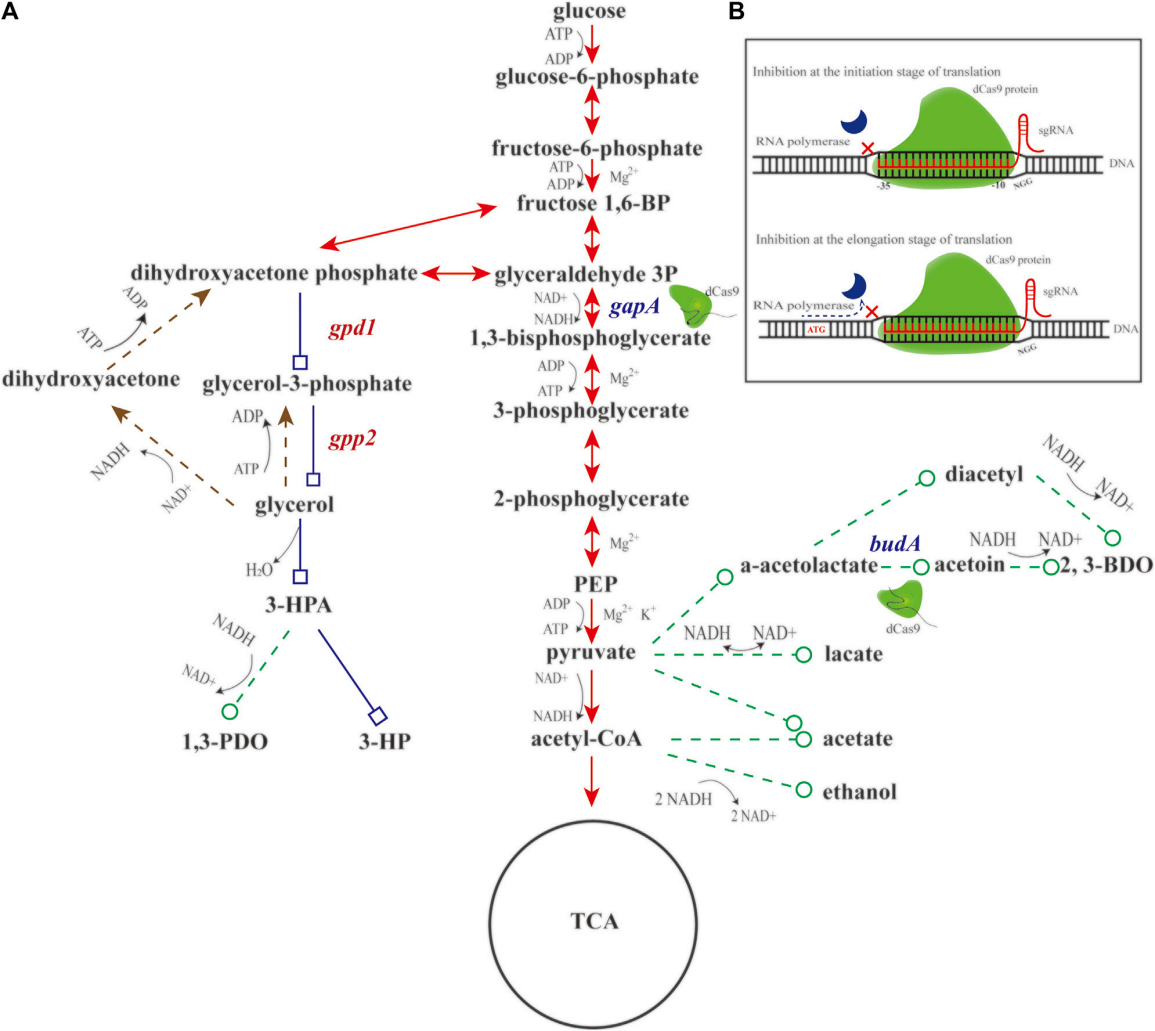
Schematic diagram of engineering glucose-to-glycerol pathway and CRISPRi system in *K. pneumoniae*. **(A)** Diagram of engineering glucose-to-glycerol pathway. **(B)** Diagram of the CRISPRi system. Triangular arrow of solid line denotes the EMP pathway. Triangular arrow of dotted line denotes the glycerol oxidation pathway. Square arrow of solid line denotes the engineered glucose to 3-hydroxypropionic acid (3-HP) pathway, and circular arrow of dotted line denotes by-product pathways. *gapA* and *budA* are two native genes inhibited by CRISPRi. *gpd1* and *gpp2* are two heterologous genes introduced for glycerol production. Fructose 1,6-BP, fructose 1,6-diphosphate; glyceraldehyde 3P, glyceraldehyde-3-phosphate; PEP, phosphoenolpyruvate; 3-HPA, 3-hydroxypropionaldehyde; 3-HP, 3-hydroxypropionic acid; 1,3-PDO, 1,3-propanediol; 2,3-BDO, 2,3-butanediol; *gpd1*, glycerol 3-phosphate dehydrogenase; *gpp2*, glycerol 3-phosphatase; *gapA*, glyceraldehyde-3-phosphate dehydrogenase; and *budA*, α-acetolactate decarboxylase.

CRISPR interference (CRISPRi, Figure 1B) is a powerful tool for modulation of multiple genes (Qi et al., 2013). In fact, fermentation titer is a quantitative trait controlled by multiple genes. The guide RNAs in the CRISPR system can direct dCas9 to user-defined genetic loci, leading to suppression of single or multiple genes and alternation of metabolites formation (Jakounas et al., 2015; Liu et al., 2019; Xu and Qi, 2019). For instance, lactic acid production can be attenuated by an engineered CRISPRi system targeting D-lactate dehydrogenase gene in *K. pneumoniae* (Wang et al., 2018). Conversely, aconitic acid production can be improved by an engineered CRISPRi system that simultaneously targeting pyruvate kinase (PK) in the glycolytic pathway (EMP pathway) and isocitrate dehydrogenase (IDH) in the tricarboxylic acid cycle (TCA cycle) pathway in *E. coli* (Li et al., 2020). These studies highlight the potential of the CRISPRi system in allocating metabolic flux.

Given aforementioned information, we conjecture that engineering a glucose-to-glycerol pathway in *K. pneumoniae* enables 3-HP production using glucose as carbon source. In addition, the CRISPRi system might be engineered to reallocate the metabolic flux toward 3-HP and other metabolites (Figure 1A). In doing so, based on the previous work, two genes, *gpd1* (GenBank accession No. Z74071.1) and *gpp2* (GenBank accession No. DQ332890.1) were used to construct the shortest pathway for conversion of glucose to 3-HP. In addition, the CRISPRi system was developed to regulate the pathways affecting 3-HP production. To boost glucose conversion to glycerol, we engineered a CRISPRi system targeting glyceraldehyde-3-phosphate dehydrogenase gene (*gapA*) and α-acetolactate decarboxylase gene (*budA*). Reverse transcription and quantitative PCR (RT-qPCR) was performed to examine the expression of *gpd1*, *gpp2*, *gapA*, and *budA*. Shake flask cultivation of the recombinant strains were to determine the production of glycerol, 3-HP, acetic acid, lactic acid, 1,3-propanediol (1,3-PDO), and 2,3-butanediol (2,3-BDO). Fed-batch cultivation of the strain was to examine the production of 3-HP.

## Materials and Methods

### Strains, Vectors, and Medium

The recombinant *K. pneumoniae* strain with dCas9 integrated in genome (KP-dCas9) and plasmid ptac-15A was preserved in laboratory (Zhao et al., 2021). The plv plasmid was provided by Professor George Guoqiang Chen from Tsinghua University (Lv et al., 2015). *E. coli* Top 10 was purchased from Biomed, China. The recombinant *K. pneumoniae* strain with dCas9 integrated in genome was used as the host strain for the development of the CRISPRi system and co-overexpression of *gpd1* and *gpp2* genes for the conversion of glucose-to-glycerol (Supplementary Table S1). *E. coli* Top 10 was employed for vector construction. The primers used in this study are listed in Supplementary Table S2. *S. cerevisiae* was used as the donor strain of genes *gpd1* and *gpp2*. The original T7 promoter in vector pET-28A was replaced by the *tac* promoter, resulting in a vector designated ptac-15A, which was used to overexpress *gpd1* and *gpp2*. In vector construction experiments, *E. coli* Top 10 was grown in Luria-Bertani (LB) medium containing the following components per liter: NaCl 10 g, tryptone 10 g, yeast extract 5 g, and chloramphenicol 34 mg. In CRISPRi and fermentation experiments, *K. pneumoniae* strain was grown in fermentation medium containing the following components per liter: glucose, 20 g; (NH_4_)_2_SO_4_, 4 g; K_2_HPO_4_·3H_2_O, 3.4 g; KH_2_PO_4_, 1.3 g; MgSO_4_·7H_2_O, 0.5 g; yeast extract, 3 g; CaCO_3_, 0.1 g; 1.25 ml of trace element solution; and chloramphenicol 34 mg. The trace element solution contained the following components per liter: CuCl_2_·2H_2_O, 1.88 g; FeSO_4_, 32 g; CoCl_2_·6H_2_O, 1.88 g; ZnCl_2_·6H_2_O, 2.72 g; Na_2_MoO_4_, 0.02 g; MnCl_2_·4H_2_O, 0.68 g; H_3_BO_3_, 0.24 g; and 40 ml concentrated HCl. Restriction enzymes, Taq DNA polymerase, and T4 DNA ligase were purchased from New England Biolabs (Beijing, China). Primer synthesis and DNA sequencing were performed by Biomed Co., Ltd. RT-qPCR was completed by RuiBiotech Co., Ltd.

### Construction of Recombinants

To overexpress GPD1 and GPP2 in KP-dCas9, their coding genes *gpd1* (GenBank accession No. Z74071.1) and *gpp2* (GenBank accession No. DQ332890.1) genes were cloned by PCR from *S. cerevisiae* genome. Next, the *gpd1* gene was cloned into ptac-15A at *Noc* I/*Nde* I sites, leading to a vector designated ptac-G1; the *gpp2* gene was cloned into ptac-15A at *Nde* I/*Nhe* I sites, leading to a vector designated ptac-G2. Ligating *gpd1* with the tac promoter and the RBS sequence from ptac-G1 to vector ptac-G2 at *Not* I/*Xho* I sites led to a vector designated ptac-G12 (Supplementary Figure S2). Transformation of vectors ptac-15A and ptac-G12 into competent KP-dCas9 cells led to recombinant strains KP-15A and KP-G12, respectively (Supplementary Table S1). KP-dCas9 was transformed with the constructed plasmid by electro-transformation (0.2 cm, 2.5 kV, and time duration >0.5 ms). *E. coli* was transformed with the constructed plasmid by heat shock. Positive clones were confirmed by colony PCR and DNA sequencing.

To develop the CRISPRi system in KP-dCas9, the CRISPRi vectors targeting *gapA* or *budA* were constructed by replacement of the sgRNA sequence in vector plv-sgRNA (Supplementary Table S1, plv-*gapA-*1, plv-*gapA-*2, plv-*gapA-*3, plv-*budA-*1, plv-*budA-*2, and plv-*budA-*3). Briefly, two complementary single-stranded target sequences were chemically synthesized and annealed to form a 23 bp double-stranded DNA owning cohesive ends matching the *Bsp*Q I-digested vector (Table 1). Subsequent ligation resulted in desired CRISPRi vectors. For each *gapA* or *budA*, three candidate guide RNAs targeting the different regions were synthesized to ensure efficient inhibition, and the sgRNA sequences, *tet* promoter and an RBS sequence from plv-vector were cloned into ptac-G12 at *Eag* I/*Bmt* I sites (Supplementary Table S1, ptac-GS, ptac-GG1, ptac-GG2, ptac-GG3, ptac-GB1, ptac-GB2, and ptac-GB3). Transforming vectors ptac-GS, ptac-GG1, ptac-GG2, ptac-GG3, ptac-GB1, ptac-GB2, and ptac-GB3 into competent KP-dCas9 led to recombinant strains KP-GS, KP-GG1, KP-GG2, KP-GG3, KP-GB1, KP-GB2, and KP-GB3, respectively (Supplementary Table S1). Last, RT-qPCR was conducted to determine the most inhibited gene. The best-performing *gapA* and *budA* sgRNAs were cloned using isocaudamers *Ngo*M IV and *Xma* I and then ligated in tandem. The sgRNA expression cassettes were digested by *Eag* I and *Ngo*M IV, while the plasmid was digested by *Eag* I and *Xma* I, leading to vector named ptac-GGB. Transforming the vector ptac-GGB into competent KP-dCas9 led to the recombinant strain KP-GGB (Supplementary Table S1; Supplementary Figure S3).

**TABLE 1:** sgRNAs used in this study.

sgRNA name	Sequence (5′–3′)	Targeting site
*budA*-sg1-F	AAATTC​GTA​AAC​CCC​GCT​CAG​CA	113–132
*budA*-sg1-R	AACTGC​TGA​GCG​GGG​TTT​ACG​AA	
*budA*-sg2-F	AAACAC​GCT​CTC​GGG​ATG​CTG​CG	65–84
*budA*-sg2-R	AACCGC​AGC​ATC​CCG​AGA​GCG​TG	
*budA*-sg3-F	AAAGTT​GCT​GGC​GGC​TCA​CCG​GA	339–358
*budA*-sg3-R	AACTCC​GGT​GAG​CCG​CCA​GCA​AC	
*gapA*-sg1-F	AAAGGT​ACA​CTC​CAC​AAT​CAC​CT	272–291
*gapA*-sg1-R	AACAGG​TGA​TTG​TGG​AGT​GTA​CC	
*gapA*-sg2-F	AAACGT​TGA​TAG​CCA​CCA​CTT​CC	81–100
*gapA*-sg2-R	AACGGA​AGT​GGT​GGC​TAT​CAA​CG	
*gapA*-sg3-F	AAAATC​GTT​GAC​GTT​GTA​GAC​GA	386–405
*gapA*-sg3-R	AACTCG​TCT​ACA​ACG​TCA​ACG​AT	

F, forward; R, reverse.

### RT-qPCR Analysis of CRISPR Interference

To examine the transcription levels of *gpd1*, *gpp2*, *gapA*, and *budA*, RT-qPCR was performed. Briefly, strains including KP-dCas9, KP-G12, KP-GS, KP-GG1, KP-GG2, KP-GG3, KP-GB1, KP-GB2, KP-GB3, and KP-GGB were cultivated in medium for 24 h and harvested by centrifugation at 4°C, 12,000 rpm. Then, the harvested bacteria were lysed with TRIzol reagent. The RNA samples were used as template to synthesize cDNAs through reverse transcription. RT-qPCR was carried out by Applied Biosystems™ 7900HT Fast Real-Time PCR System (Thermo Fisher) with SYBR Green addition. The RT-qPCR data were analyzed using the ΔΔCT method with 16S rRNA as an internal control. All experiments were performed in triplicate.

### Shake Flask and Bioreactor Cultivation

In shake flask cultivation, strains KP-dCas9, KP-15A, KP-GS, KP-GG1, KP-GB2, and KP-GGB were pre-cultured in 4 ml LB medium overnight. Next, these strains were independently grown in 250-ml shake flasks each containing 100 ml 3-HP–producing medium and 8.5 mg chloramphenicol at 37°C and 150 rpm. After 3 h cultivation, IPTG at a final concentration of 0.5 mM was added to induce the expression of *gpd1* and *gpp2*. Dehydrated tetracycline (aTc) at a final concentration of 2 μM was added to induce sgRNA expression. Fermentation broth was sampled every 3 h to examine cell growth, glucose consumption, and metabolites formation.

In fed-batch cultivation, the recombinant strain was grown in a 5 L bioreactor (Baoxing, China) containing 3 L fermentation medium to produce 3-HP. The strain KP-GB2 was pre-cultured in 100 ml of LB medium overnight at 37°C and subsequently inoculated into a bioreactor, containing antibiotics and IPTG. Air was supplied at 1.5 vvm, and agitation speed was set at 400 rpm. The temperature was set at 37°C, and the pH was maintained at 7.0 by adding 5 M NaOH. A total of 700 ml of 500 g/L glucose solution was added at a rate of 4.5 g/h to maintain cell growth after the glucose in medium was exhausted. The samples were taken out every 6 h to examine cell growth, glucose consumption, and metabolites formation.

### Analytical Methods

Cell concentrations were measured by using microplate reader at 600 nm with 200 μL fermentation broth and 1,800 μL ddH_2_O added in a cuvette. To measure metabolites, fermentation broth was centrifuged at 12,000 rpm for 10 min to remove bacteria. Major metabolites including 3-HP, lactic acid, and acetic acid in supernatant were analyzed by using the high-performance liquid chromatography (HPLC) system (Shimadzu, Kyoto, Japan) equipped with a C_18_ column and an SPD-20A UV detector at 210 nm. The column was maintained at 25°C. The mobile phase was 0.05% phosphoric acid at a flow rate of 0.8 ml/min. 1,3-PDO and 2,3-BDO were analyzed by GC (Persee). Analytically pure 1,3-PDO and 2,3-BDO were used as standard for quantification. Residual glucose concentration was measured by an SBA-40E biosensor analyzer (Biology Institute of Shandong Academy of Science, China). A new immobilized enzyme membrane was installed on the electrode, 500 ml standard buffer was prepared with ddH_2_O, and the three-in-one standard solution of SBA was prepared (three materials mentioned earlier are provided by the instrument manufacturer). Glucose concentration in the supernatant was diluted to less than 1 g/L. After 25 μL standard solution was injected into the sampling port stabilization instrument with a microinjector, the glucose content in the sample was measured successively, and each sample was measured three times. Glycerol concentration was measured by the glycerin test kit (APPLYGEN Beijing). All samples were filtered through a 0.22 μm membrane filter.

## Result

### Determination of Plasmids

**FIGURE 2:**
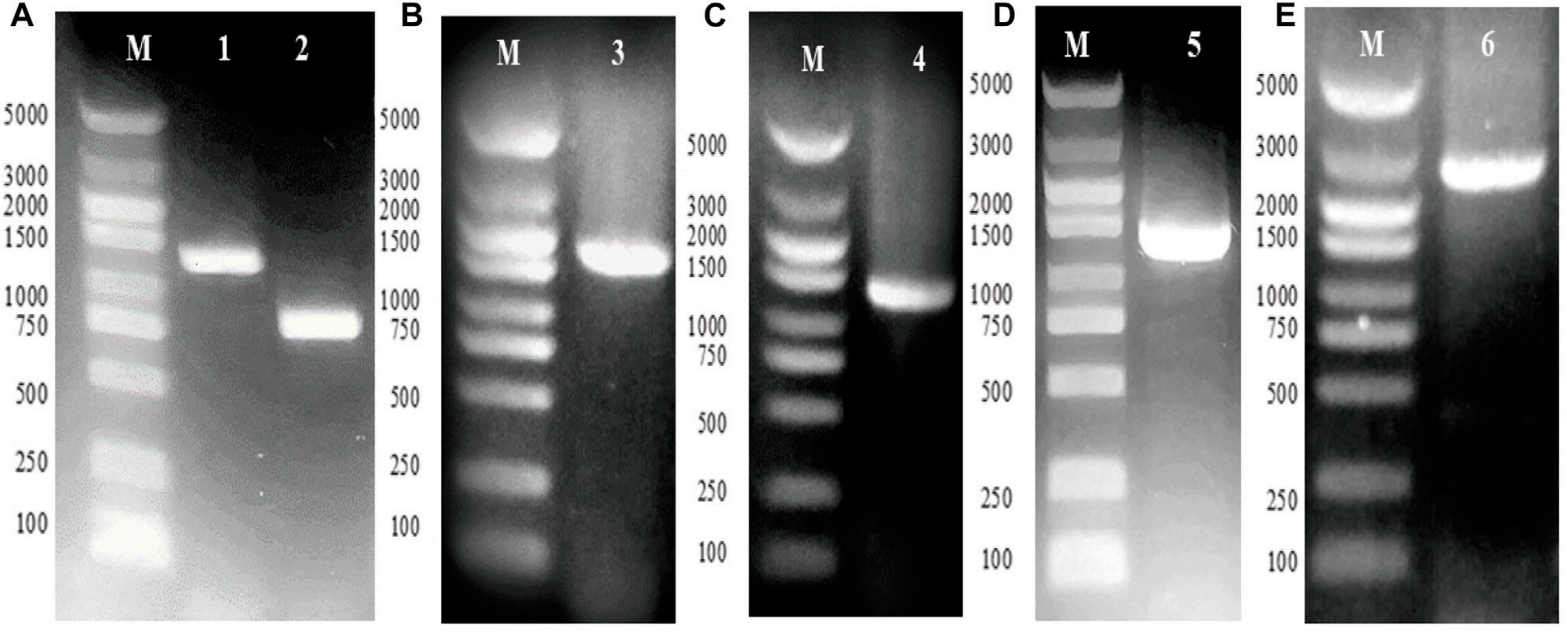
Construction of plasmid ptac-G12 that co-expressing *gpd1* and *gpp2* under tac promoter. G1 denotes *gpd1* gene encoding glycerol 3-phosphate dehydrogenase; G2 denotes *gpp2* gene encoding glycerol 3-phosphatase; and G12 denotes co-expression of *gpd1* and *gpp2*. **(A)** PCR amplification of the *gpd1* and *gpp2* genes from *S. cerevisiae*. **(B)** Colony PCR of plasmid ptac-*gpd1*. **(C)** Colony PCR of plasmid ptac-*gpp2*. **(D)** PCR amplification of ptac-*gpd1* expression cassette. **(E)** Colony PCR of *gpd1* and *gpp2.* M, Marker; lane 1, *gpd1* gene; lane 2, *gpp2* gene; lane 3, plasmid ptac-*gpd1* expression cassette; lane 4, plasmid ptac-*gpp2* expression cassette; lane 5, plasmid ptac-*gpd1* expression cassette; and lane 6, colony PCR of the co-expressed *gpd1* and *gpp2* in tandem.

The genes *gpd1* and *gpp2* were cloned from *S. cerevisiae* and independently subcloned into vector ptac-15A, resulting in vectors ptac-G1 and ptac-G2, respectively (Figures 2A–C). Next, PCR was conducted to obtain ptac-G1 as template, *gpd1* fragment, *tac* promoter, and operon (Figure 2D). These fragments were then inserted into ptac-G2 to construct ptac-G12 (Figure 2E). The two genes *gpd1* and *gpp2* were expressed using respective *tac* promoter, and their expression was induced by IPTG.

### Glycerol Production by Engineered *K. pneumoniae*

**FIGURE 3:**
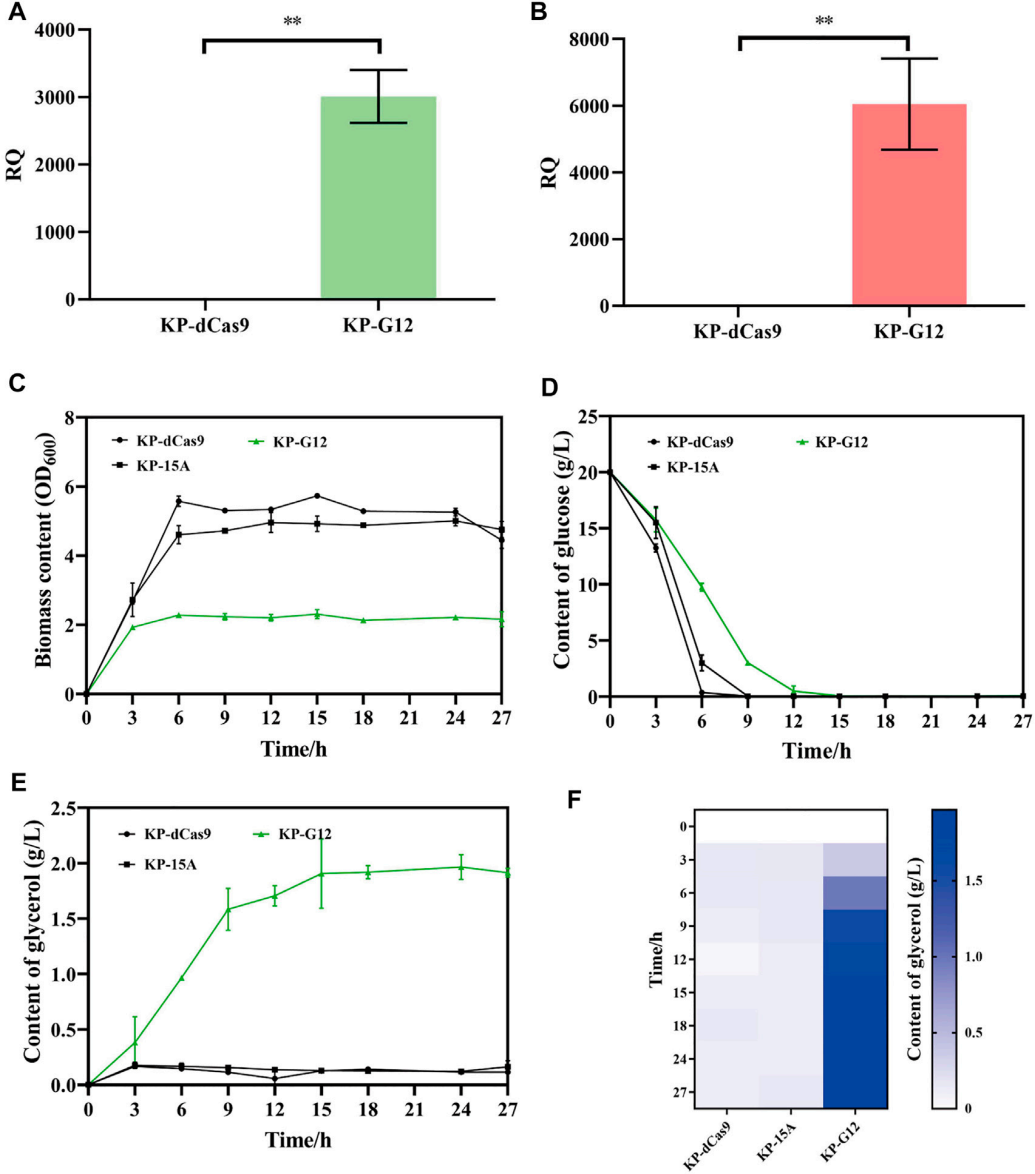
Performance of strains in shake flasks. **(A)** Relative quantity of *gpd1* gene transcription. **(B)** Relative quantity of *gpp2* gene transcription. KP-dCas9 indicates the recombinant *K. pneumoniae* with dCas9 integrated in genome, and its 16S rRNA serves as the internal reference gene. **(C)** Glucose level after 24 h fermentation. **(D)** OD_600_ after 24 h fermentation. **(E)** Line chart represents the glycerol level. **(F)** Heatmap of the glycerol level. KP-dCas9 indicates the recombinant *K. pneumoniae* with dCas9 integrated in genome. KP-15A indicates the recombinant *K. pneumoniae* harboring plasmid ptac-15A and serves as a control. KP-G12 indicates the recombinant *K. pneumoniae* harboring plasmid ptac-G12 co-expressing *gpd1* and *gpp2* genes. RQ, relative quantity of transcription. *p < 0.05 and **p < 0.01.

The recombinant *K. pneumoniae* strain was cultured in a shake flask to verify glycerol production. To construct glucose-to-glycerol pathway, *gpd1* and *gpp2* were co-expressed in strain KP-dCas9, which harbored the engineered CRISPRi system. In doing so, we constructed a recombinant strain named KP-G12, and the strains KP-15A and KP-dCas9 were used as controls. RT-qPCR results showed that the transcription levels of *gpd1* and *gpp2* were significantly increased in KP-G12 compared to KP-dCas9 (Figures 3A,B). In 24 h cultivation, glucose consumption was slowed down due to the expression of heterologous genes (Figure 3C), and the strain KP-G12 showed a significant decrease in OD_600_ (Figure 3D). The strain containing vector ptac-G12 produced 2 g/L glycerol, with 0.1 g/g yield on glycerol (g of glucose)^−1^. In contrast, no glycerol was produced by strains KP-dCas9 and KP-15A (Figure 3E,F), indicating that *gpd1* and *gpp2* catalyzed glucose into glycerol in *K. pneumoniae*.

### CRISPR Interference System Inhibiting EMP Downstream Pathway and 2,3-BDO Production

**FIGURE 4:**
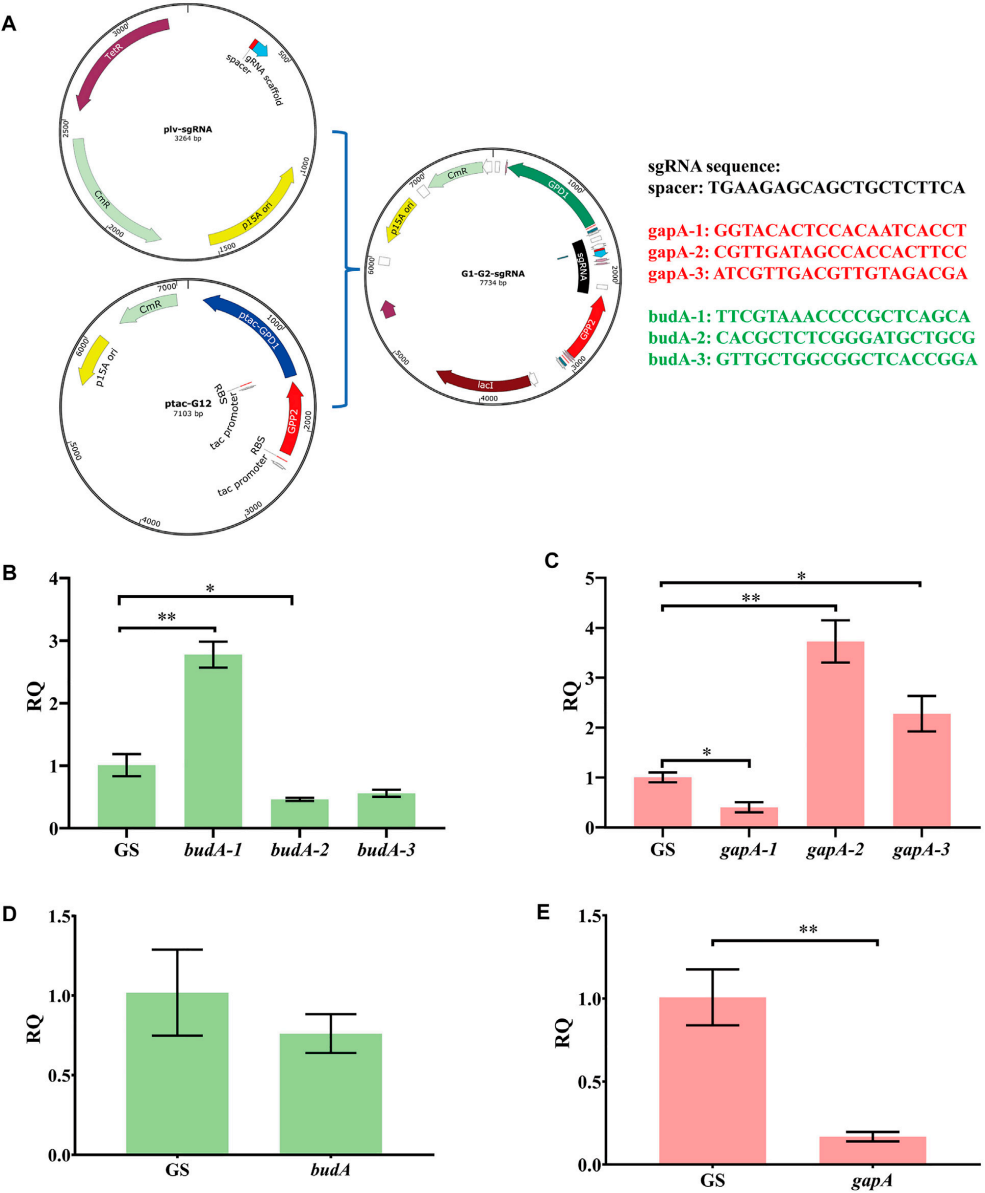
Relative transcription quantity of *K. pneumoniae* with dCas9 integrated in genome and an engineered CRISPRi system. **(A)** Schematic diagram of linking sgRNA to plasmid ptac-G12 co-expressing *gpd1* and *gpp2*. **(B)** RT-qPCR analysis of CRISPRi inhibition on different regions of the *budA* gene. *budA*-1, *budA*-2, and *budA*-3 represent different regions of *budA* gene. GS, recombinant *K. pneumoniae* harboring the non-targeting CRISPRi system. **(C)** RT-qPCR analysis of CRISPRi inhibition on different regions of *gapA* gene. *gapA*-1, *gapA*-2, and *gapA*-3 represent different regions of the *budA* gene. **(D)** RT-qPCR analysis of transcription inhibition on the *budA* gene by the CRISPRi system carrying the sgRNAs for both *budA* and *gapA*. **(E)** RT-qPCR analysis of transcription inhibition on the *gapA* gene by the CRISPRi system carrying the sgRNAs for both *budA* and *gapA*. *gapA*-1, *gapA*-2, and *gapA*-3 denote different regions of the *gapA* gene. RQ, relative quantity of transcription. *p < 0.05 and **p < 0.01.

To optimize the CRISPRi system, we prepared several guide RNAs named G1, G2, G3, B1, B2, and B3 that target the different regions of *gapA* and *budA* (Supplementary Figures S1A,B,G). The aforementioned six sgRNAs and one non-target sgRNA were independently ligated to vector ptac-G12 (Supplementary Figures S1C,D; Figure 4A). Transforming these vectors into KP-dCas9 led to recombinant strains KP-GG1, KP-GG2, KP-GG3, KP-GB1, KP-GB2, KP-GB3, and KP-GS, respectively. The strain KP-GS was used as the control. RT-qPCR results showed that compared to KP-GS, the strains KP-GG1 and KP-GB2 showed significant decrease in the expression of target genes (Figures 4A,B). The *gapA* gene in strain KP-GG1was transcriptionally inhibited by 60%, and the *budA* gene in strain KP-GB2 was transcriptionally inhibited by 54%. When *budA*-2 was connected in series with *gapA*-1, *gpd1*, and *gpp2* (Supplementary Figures S1E,F), the resulting plasmid was named GGB, and the corresponding recombinant strain was named KP-GGB. In strain KP-GGB, the inhibitory rates of the CRISPRi system on genes *gapA* and *budA* reached 82 and 24%, respectively.

### Shake Flask Cultivation of the CRISPR Interference Strains

To maximize 3-HP and minimize 2,3-BDO, the glycerol synthesis genes *gpd1* and *gpp2* were co-expressed, and two genes *gapA* and *budA* were inhibited by the CRISPRi system. RT-qPCR results showed that the strains KP-GG1, KP-GB2, and KP-GGB demonstrated the strongest CRISPR inhibition under micro-oxygen conditions as compared to the strains KP-GS, KP-dCas9, and KP-15A. In 24 h cultivation, these engineered strains showed retarded cell growth and reduced glucose consumption. All engineered *K. pneumoniae* strains produced glycerol, but the difference was not significant. For 2,3-BDO production, the strain KP-15A presented the highest titer (8.39 ± 0.33 g/L), while the strain KP-GB2 presented the lowest titer (1.185 ± 0.045 g/L). In addition, the strain KP-GB2 produced the highest 3-HP (0.734 ± 0.032 g/L). Interestingly, no significant differences were observed in the production of acetic acid, lactic acid, and 1,3-PDO in all strains.

### Fed-Batch Cultivation of CRISPR Interference Strains

Now that the strain co-expressing *gpd1* and *gpp2* was able to synthesize glycerol from glucose and the strain KP-GB2 showed the highest 3-HP level in the shake flask, the strain was then cultivated in a 5 L bioreactor. Results showed that the strain did not consume glycerol until 12 h fermentation, at this time the glucose was exhausted. When glucose was supplemented, the glycerol level was further improved, indicating that the strain did not consume glycerol until glucose was exhausted. In addition, only a small amount of 2,3-BDO was produced in the initial stage of fermentation. However, in the middle and late stages of fermentation, 2,3-BDO production was increased presumably due to the rising pH value of medium and the failure of aTc induction in an alkaline environment, which hampered the inhibition of the CRISPRi system on by-product pathways. Due to the engineered glycerol synthesis pathway, the strain KP-GB2 produced 1.77 ± 0.04 g/L 3-HP in 72 h, with productivity of 0.025 g/L/h. This low productivity may be ascribed to the engineered CRISPRi system and genes *gpd1* and *gpp2*, which imposed a heavy burden on cell growth. Indeed, the maximum OD_600_ was only 12.135 ± 0.005.

## Discussion

**FIGURE 5:**
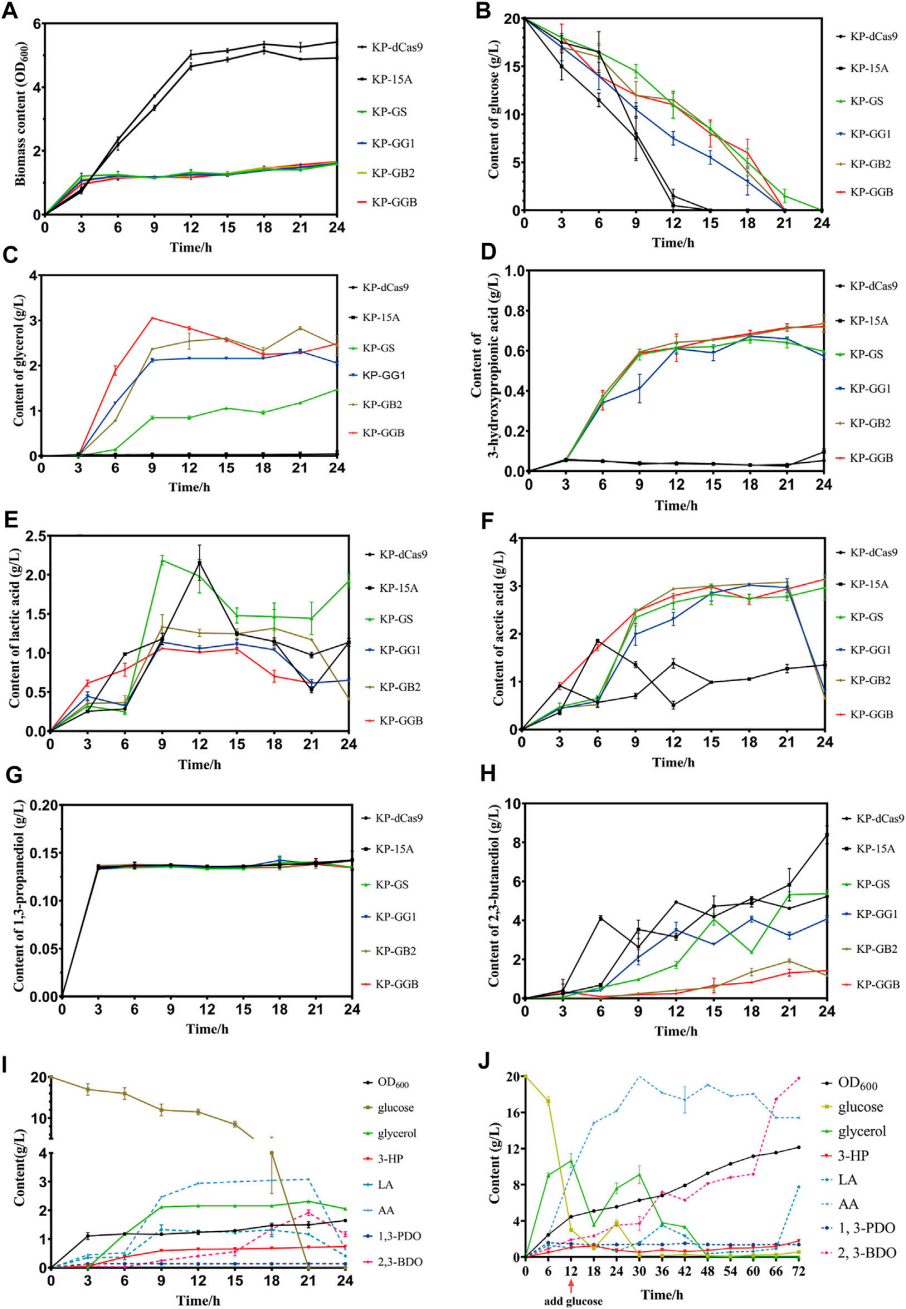
Fermentation of strain harboring CRISPRi system to repress by-product pathways. **(A)** Biomass (OD_600_) of the CRISPRi strain in the shake flask. **(B)** Glucose consumption of CRISPRi strain in the shake flask. **(C)** Glycerol production by CRISPRi strain in the shake flask. **(D)** 3-Hydroxypropionic acid production by CRISPRi strain in the shake flask. **(E)** Lactic acid production by CRISPRi strain in the shake flask. **(F)** Acetic acid production by CRISPRi strain in the shake flask. **(G)** 1,3-Propanediol production of CRISPRi strain in the shake flask. **(H)** 2,3-Butanediol production by CRISPRi strain in the shake flask. **(I)** Metabolites of CRISPRi strain KP-GB2 in the shake flask. **(J)** Metabolites of CRISPRi strain KP-GB2 in fed-batch culture. KP-dCas9, the recombinant *K. pneumoniae* strain with dCas9 integrated in genome; KP-15A, the recombinant *K. pneumoniae* strain harboring empty vector ptac-15A; KP-GS, the recombinant *K. pneumoniae* with dCas9 integrated in genome, and harboring vector ptac-GS co-expressing non-target sgRNA, *gpd1* and *gpp2*; KP-GG1, recombinant *K. pneumoniae* with dCas9 integrated in genome of and ptac-GG1 plasmid co-expressing *gpd1*, *gpp2*, and *gapA-*targeting sgRNA; KP-GB2, the recombinant *K. pneumoniae* with dCas9 integrated in genome and vector ptac-GB2 co-expressing *gpd1*, *gpp2*, and *budA-*targeting sgRNA; KP-GGB, the recombinant *K. pneumoniae* with dCas9 integrated in genome and plasmid ptac-GGB co-expressing *gpd1*, *gpp2*, and sgRNAs targeting *gapA* and *budA*. 3-HP, 3-hydroxypropionic acid; LA, lactic acid; AA, acetic acid; 1,3-PDO, 1,3-propanediol; and 2,3-BDO, 2,3-butanediol.

In this study, the glycerol synthesis pathway was engineered in *K. pneumoniae*, and the resulting strain was subjected to CRISPRi-dependent gene regulation (Figure 2). By co-expressing *gpd1* and *gpp2* and simultaneously inhibiting *gapA* and *budA*, the levels of both glycerol and 3-HP were improved (Figure 4, Figure 5). In addition, the by-products and glucose flux in the EMP pathway were reduced. The aforementioned results confirmed the engineered glucose to the 3-HP pathway in *K. pneumoniae*. In *K. pneumoniae*, glycerol-based 3-HP synthesis requires only two reactions, and the theoretical conversion rate is therefore high. After introducing *gpd1* and *gpp2* into *K. pneumoniae*, glucose-based production of 3-HP requires only four enzyme genes including *gpd*, *gpp2*, *gdh*, and *aldH* (e.g., *puuC*). As for the low 3-HP production, it was ascribed to the presence of glucose. In particular, the Crabtree effect hindered glycerol utilization. Future studies may focus on how to attenuate the Crabtree effect. To address this issue, an orthogonal expression system could be engineered to express glycerol utilization genes and minimize the Crabtree effect so that glycerol can be efficiently converted to 3-HP while cell growth is not compromised. Clearly, profound understanding of the *dha* regulon and Crabtree effect is a prerequisite for achieving this goal.

The Crabtree effect and Pasteur effect (Winkler et al., 2003) are two important regulatory mechanisms affecting glucose metabolism. The Crabtree effect is a common problem encountered by mixed carbon source fermentation. Removal of the Crabtree effect enables bacteria to co-utilize glucose and other carbon sources. The Pasteur effect entangles the choice of aeration during fermentation. Aerobic respiration increases bacterial growth but reduces the activity of phosphofructokinase through feedback inhibition, thereby inhibiting the EMP pathway. In fed-batch cultivation, it is necessary to maintain different oxygen levels in bioreactor for different purposes. If the Crabtree effect can be attenuated and aeration can be adjusted in fed-batch cultivation, the strain will use glucose as a carbon source for growth, which will in turn improve the production of glycerol and 3-HP. However, it is extremely challenging to completely decouple glucose pathways from glycerol pathways, and co-utilization of different carbon sources may be feasible to maintain active cell growth.

CRISPRi is usually not lethal to host cell, as it does not digest DNA. In contrast, gene knockout tools such as CRISPR editing, RecA homologous recombination, and Red homologous recombination are in most cases lethal to host cells. In present study, CRISPRi was developed to inhibit *gapA*, an enzyme catalyzes 3-glyceraldehyde triphosphate into 1,3-diphosphoglyceric acid. CRISPRi was also used to inhibit *budA*, an enzyme required for 2,3-BDO production in *K. pneumoniae* (Yang et al., 2014). RT-qPCR results showed that the engineered CRISPRi systems could significantly inhibit the expression of *budA* and *gapA*. In shake flask cultivation, the CRISPRi strain demonstrated slight enhancement in the glycerol level but significant decrease in 2,3-BDO production. However, the inhibitory effect on *bud*A was significantly reduced when two groups of sgRNAs were linked in tandem. This might be ascribed to the low expression of dCas9, which was insufficient for binding two target genes simultaneously. Plasmid-based overexpression of dCas9 and multi-copy integration of dCas9 into genome may be feasible solutions. Overall, this study demonstrated the feasibility of glucose-to-glycerol as well as CRISPRi-dependent gene regulation in *K. pneumoniae*. We anticipate that better understanding of the metabolic networks is crucial for high-level production of 3-HP, 1,3-PDO, 2,3-BDO, and beyond.

## Data Availability

The original contributions presented in the study are included in the article/Supplementary Material; further inquiries can be directed to the corresponding author.
