# Supplementary material for: Engineering Glucose-to-Glycerol Pathway in Klebsiella pneumoniae and Boosting 3-Hydroxypropionic Acid Production Through CRISPR Interference
Source: Front Bioeng Biotechnol. 2022 Jun 30;10:908431. doi: 10.3389/fbioe.2022.908431 (PMC9280265; doi:10.3389/fbioe.2022.908431)
Supplement: Supplementary file 1 [file DataSheet1.docx]

**Table S1 Vectors and strains used in this study**

| Vectors and strains | Description | Source |
| --- | --- | --- |
| **vectors** |  |  |
| p*tac*-15A | The original T7 promoter in pET-28A was replaced by *tac* promoter, Cm^R^ | Lab preservation |
| plv | CRISPRi vector, sgRNA under *tet* promoter, Cm^R^ | Tsinghua University |
| p*tac*-*gpd1* | Recombinant vector overexpressing the *gpd1* gene from *S. cerevisiae*, *tac* promoter, Cm^R^ | This study |
| p*tac*-*gpp2* | Recombinant vector overexpressing the *gpp2* gene from *S. cerevisiae*, *tac* promoter, Cm^R^ | This study |
| p*tac*-G12 | Recombinant vector coexpressing *gpd1* and *gpp2* genes from *S. cerevisiae*, *tac* promoter, Cm^R^ | This study |
| plv-*gapA*1 | CRISPRi vector targeting *gapA* gene, Cm^R^ | This study |
| plv-*gapA*2 | CRISPRi vector targeting *gapA* gene, Cm^R^ | This study |
| plv-*gapA*3 | CRISPRi vector targeting *gapA* gene, Cm^R^ | This study |
| plv-*budA*1 | CRISPRi vector targeting *budA* gene, Cm^R^ | This study |
| plv-*budA*2 | CRISPRi vector targeting *budA* gene, Cm^R^ | This study |
| plv-*budA*3 | CRISPRi vector targeting *budA* gene, Cm^R^ | This study |
| p*tac*-GS | CRISPRi vector with spacer (non-target) sgRNA, overexpressing *gpd1* and *gpp2* genes from *S. cerevisiae*, *tac* promoter, Cm^R^ | This study |
| p*tac*-GG1 | CRISPRi vector targeting *gapA* gene and overexpressing *gpd1* and *gpp2* genes from *S. cerevisiae*, *tac* promoter, Cm^R^ | This study |
| p*tac*-GG2 | CRISPRi vector targeting *gapA* gene and overexpressing the *gpd1* and *gpp2* genes from *S. cerevisiae*, *tac* promoter, Cm^R^ | This study |
| p*tac*-GG3 | CRISPRi vector targeting *gapA* gene and overexpressing the *gpd1* and *gpp2* genes from *S. cerevisiae*, *tac* promoter, Cm^R^ | This study |
| p*tac*-GB1 | CRISPRi vector targeting *budA* gene and overexpressing the *gpd1* and *gpp2* genes from *S. cerevisiae*, *tac* promoter, Cm^R^ | This study |
| p*tac*-GB2 | CRISPRi vector targeting *budA* gene and overexpressing the *gpd1* and *gpp2* genes from *S. cerevisiae*, *tac* promoter, Cm^R^ | This study |
| p*tac*-GB3 | CRISPRi vector targeting *budA* gene and overexpressing the *gpd1* and *gpp2* genes from *S. cerevisiae*, *tac* promoter, Cm^R^ | This study |
| p*tac*-GGB | CRISPRi vector targeting *gapA* and *budA* genes and overexpressing the *gpd1* and *gpp2* genes from *S. cerevisiae*, *tac* promoter, Cm^R^ | This study |
| **strains** |  |  |
| *E. coli* TOP10 | Competent cell used for vector construction | Biomed, China |
| EC-15A | *E. coli* TOP10 harboring empty vector p*tac*-15A | This study |
| EC-G1 | *E. coli* TOP10 harboring vector p*tac*-*gpd1* | This study |
| EC-G2 | *E. coli* TOP10 harboring vector p*tac*-*gpp2* | This study |
| EC-G12 | *E. coli* TOP10 harboring vector p*tac*-G12 | This study |
| EC-G1 | *E. coli* TOP10 harboring vector plv-*gapA*1 | This study |
| EC-G2 | *E. coli* TOP10 harboring vector plv-*gapA*2 | This study |
| EC-G3 | *E. coli* TOP10 harboring vector plv-*gapA*3 | This study |
| EC-B1 | *E. coli* TOP10 harboring vector plv-*budA*1 | This study |
| EC-B2 | *E. coli* TOP10 harboring vector plv-*budA*2 | This study |
| EC-B3 | *E. coli* TOP10 harboring vector plv-*budA*3 | This study |
| EC-GS | *E. coli* TOP10 harboring vector ptac-GS | This study |
| EC-GG1 | *E. coli* TOP10 harboring vector ptac-GG1 | This study |
| EC-GG2 | *E. coli* TOP10 harboring vector ptac-GG2 | This study |
| EC-GG3 | *E. coli* TOP10 harboring vector ptac-GG3 | This study |
| EC-GB1 | *E. coli* TOP10 harboring vector ptac-GB1 | This study |
| EC-GB2 | *E. coli* TOP10 harboring vector ptac-GB2 | This study |
| EC-GB3 | *E. coli* TOP10 harboring vector ptac-GB3 | This study |
| EC-GGB | *E. coli* TOP10 harboring vector ptac-GGB | This study |
| KP-dCas9 | *K. pneumoniae* with dCas9 integrated in genome | Laboratory preservation |
| KP-15A | KP-dCas9 harboring vector p*tac*-15A | This study |
| KP-G12 | KP-dCas9 harboring vector p*tac*-G12 | This study |
| KP-GS | KP-dCas9 harboring vector p*tac*-GS | This study |
| KP-GG1 | KP-dCas9 harboring vector p*tac*-GG1 | This study |
| KP-GG2 | KP-dCas9 harboring vector p*tac*-GG2 | This study |
| KP-GG3 | KP-dCas9 harboring vector p*tac*-GG3 | This study |
| KP-GB1 | KP-dCas9 harboring vector p*tac*-GB1 | This study |
| KP-GB2 | KP-dCas9 harboring vector p*tac*-GB2 | This study |
| KP-GB3 | KP-dCas9 harboring vector p*tac*-GB3 | This study |
| KP-GGB | KP-dCas9 harboring vector p*tac*-GGB | This study |

**Table S2 Primers used in this study**

| Name | Nucleotide sequence (5’–3’) |
| --- | --- |
| *gpd1*-F-*Nco* I | CATGCCATGGATGTCTGCTGCTGCTGATAG |
| *gpd1*-R-*Nde* I | CGCCATATGCTAATCTTCATGTAGATCTAATTCTTCAATCATG |
| *gpp2*-F-*Nde* I | GGAATTCCATATGATGGGATTGAC*TAC*TAAACCT |
| *gpp2*-R-*Nhe* I | CTAGCTAGCT*TAC*CATTTCAACAGATCGTCCTTAG |
| *tacgpd1*-F-*Not* I | ATAAGAATGCGGCCGCTTGACAATTAATCATCGGCT |
| *tacgpd1*-R-*Xho* I | CCGCTCGAGCTAATCTTCATGTAGATCTAATTCTTCAATCATGTCC |
| *gapA*-1F | AAAGGTACACTCCACAATCACCT |
| *gapA*-1R | AACAGGTGATTGTGGAGTGTACC |
| *gapA*-2F | AAACGTTGATAGCCACCACTTCC |
| *gapA*-2R | AACGGAAGTGGTGGCTATCAACG |
| *gapA*-3F | AAAATCGTTGACGTTGTAGACGA |
| *gapA*-3R | AACTCGTCTACAACGTCAACGAT |
| *budA*-1F | AAATTCGTAAACCCCGCTCAGCA |
| *budA*-1R | AACTGCTGAGCGGGGTTTACGAA |
| *budA*-2F | AAACACGCTCTCGGGATGCTGCG |
| *budA*-2R | AACCGCAGCATCCCGAGAGCGTG |
| *budA*-3F | AAAGTTGCTGGCGGCTCACCGGA |
| *budA*-3R | AACTCCGGTGAGCCGCCAGCAAC |
| sg-F | TGTTTGTCGGTGAACGCTCTCTACTAG |
| sg-R-*Bmt* I | CGCGCTAGCGCGGAATATATCCCTAGGCCTGCAG |
| sg-R | GCGGAATATATCCCTAGGCCTGCAG |
| FF | CACCATACCCACGCCGAAACAAGCG |
| RR | ATCCGGATATAGTTCCTCCTTTCAGCAAAAAACC |

The underlined sequences indicate restriction sites. F, forward; R, reverse.

*gapA*-1, *gapA*-2 and *gapA*-3 indicate three candidate CRISPRi vectors targeting different regions of gene *gapA* encoding glyceraldehyde 3-phosphate dehydrogenase. *budA*-1, *budA*-2 and *budA*-3 indicate three candidate CRISPRi vectors targeting different regions of gene *budA* encoding α-acetolactate decarboxylase.

sg-F and sg-R are universal primers for plv plasmid; FF and RR are universal primers for ptac-15A plasmid. They were used for colony PCR and DNA sequencing.

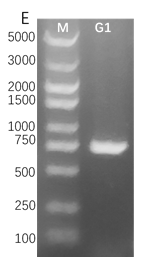

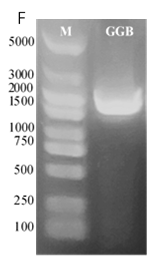

**Figure S1** Construction of sgRNAs. **A.** Colony PCR of sgRNA construction; **B.** Colony PCR of sgRNA expression cassette; **C.** Colony PCR of G12-sgRNA; **D.** Colony PCR of G12-sgRNA electro-transformed into *K. pneumoniae*; **E.** Colony PCR of sgRNA-G1 expression cassette; **F.** Colony PCR of G12-G1-B2; **G.** sgRNAs sequencing results. M, Marker; spacer, non-target sgRNA; G1-G3 denotes plv-sg-*gapA*-1to plv-sg-*gapA*-3; B1-B3 denotes plv-sg-*budA*-1to plv-sg-*budA*-3; GS indicates G1G2-spacer; GG1-GG3 indicates G1G2-*gapA*-1 to G1G2-*gapA*-3; GB1-GB3 indicates G1G2-*budA*-1 to G1G2-*budA*-3; GGB indicates G1-G2-*gapA*-1-*budA*-2.


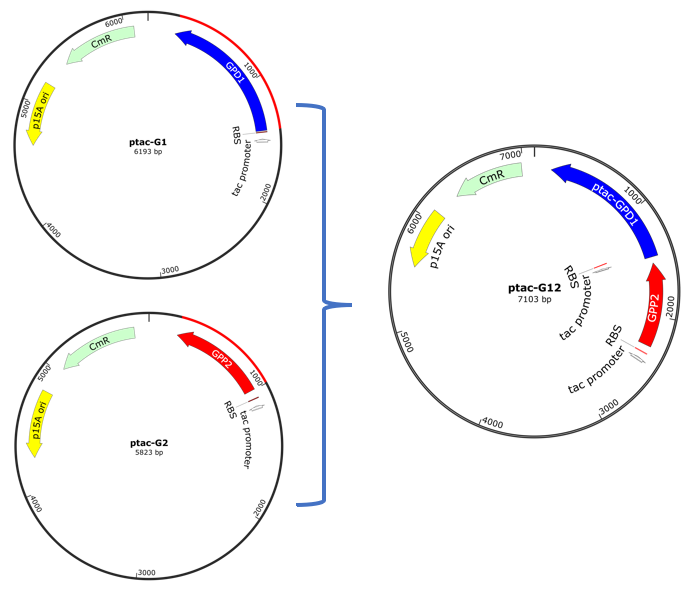


**Figure S2** Schematic diagram of linking ptac-*gpd1* to *gpp2* expression plasmid.


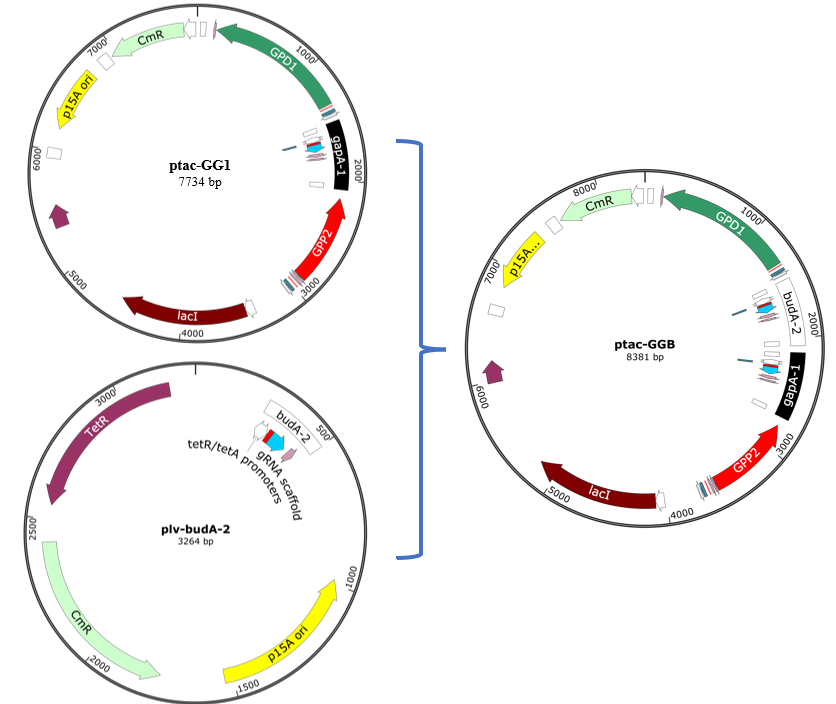


**Figure S3** Schematic diagram of linking *budA-2* to plasmid ptac-GG1.
